# Psychometric performance of the WHO-5 well-being index in a nationwide sample of inpatients discharged from specialised mental health care

**DOI:** 10.1007/s11136-025-04104-9

**Published:** 2025-12-29

**Authors:** Hilde Hestad Iversen, Marte Karoline Råberg Kjøllesdal, Lina Harvold Ellingsen-Dalskau, Mona Haugum, Oyvind Bjertnaes

**Affiliations:** 1 Norwegian Institute of Public Health, PO Box 222, 0213 Skoyen, Oslo, Norway; 2 Norwegian University of Life Sciences, PO Box 5003, 1432 Ås, Norway; 3 Center for Evidence-Based Public Health: A Joanna Briggs Institute Affiliated Group, PO Box 5003, 1433 Ås, Norway

**Keywords:** WHO-5, Well-being, Patient-reported outcome measure, Validation, Psychometrics, Mental health, Post-discharge psychiatric care

## Abstract

**Purpose:**

To evaluate the reliability and construct validity of the five-item World Health Organization Well-Being Index (WHO-5) in a nationwide sample of adults recently discharged from specialised inpatient mental health care in Norway.

**Methods:**

A total of 2310 patients participated in the national survey in early 2024. The WHO-5 was assessed for missing data, floor effects, internal consistency, and factor structure using exploratory and confirmatory factor analysis (EFA, CFA). Measurement invariance across sex, age, and education was tested with multi-group CFA. Construct validity was evaluated through correlations with subjective well-being, self-reported mental and physical health, and treatment-related enablement. Item response theory (IRT) analyses assessed item discrimination and thresholds.

**Results:**

Item-level missingness was low (<1%), with moderate to high floor effects for two items. Internal consistency reliability was high (Cronbach’s alpha: 0.910). EFA supported a one-factor solution explaining 71.7% of the variance, confirmed by CFA; fit indices were strong for CFI/TLI/SRMR, while RMSEA was elevated, consistent with very low degrees of freedom in a five-item model. Configural, metric, and scalar invariance were supported across sex, age, and education. WHO-5 correlated strongly with life satisfaction, meaning in life, and self-perceived mental health, and moderately with physical health and treatment-related enablement. IRT indicated adequate to high discrimination; thresholds were ordered but closely spaced in the mid-range, and coverage primarily spanned low to moderate levels of well-being.

**Conclusion:**

The WHO-5 demonstrated strong reliability, clear unidimensionality, and supported invariance in this population, supporting its use as a brief, generic PROM in mental health care. Observed floor effects and overlapping mid-range categories suggest scope for response-format refinement to improve measurement precision.

**Supplementary Information:**

The online version contains supplementary material available at 10.1007/s11136-025-04104-9.

## Background

Mental health conditions are among the leading causes of health loss worldwide [1], and the number of individuals reporting mental health problems has risen in recent years, increasing pressure on health services [2]. Although treatment can reduce much of this burden [1], quality measurement in mental health care still lags behind that of somatic health services, especially in evaluating service effectiveness [3]. Valid and reliable tools are needed to capture patient-reported outcomes and experiences, which are key to patient-centred care and service improvement.

Patient-reported experience measures (PREMs) and patient-reported outcome measures (PROMs) provide insight into how patients perceive their care and outcomes. These measures complement clinical indicators by giving voice to patients, supporting shared decision-making, quality improvement, and policy development [4]. As such, PREMs and PROMs are central to delivering high-quality, patient-centred services.

In Norway, the Norwegian Institute of Public Health (NIPH) has conducted extensive research on PREMs and PROMs to support a more patient-centred health system. This work includes both methodological investigations into instrument validity and reliability, and broader studies on predictors of patient experiences and social inequalities. While most previous research has focused on PREMs, the shift from cross-sectional to longitudinal data collection in mental health and substance dependence surveys has increased the relevance of PROMs [5]. Longitudinal designs allow for a deeper understanding of how patient experiences and outcomes evolve over time, particularly after discharge.

This perspective enables the tracking of changes in health status and care trajectories, supporting a more comprehensive assessment of care quality and patient well-being. Although validated PREM instruments have long been in use, the integration of PROMs is now gaining importance [6, 7]. Such integration has become a growing international priority, as reflected in the OECD’s Patient-Reported Indicator Surveys (PaRIS) initiative, which recently developed and field-tested PaRIS Patient Questionnaire (PaRIS-PQ) integrating PROMs and PREMs [4, 8–11]. PROMs capture health outcomes from the patient’s perspective, including how these relate to treatment, and can be either condition-specific or generic, covering a broad range of psychosocial and physical domains [4]. As mental illness often impacts well-being and quality of life, generic PROMs are particularly valuable in mental health care.

As part of the development of the national post-discharge survey targeting adults recently discharged from specialised inpatient mental health care, NIPH conducted a structured selection process to identify suitable PROMs for integration. Several PROMs were considered during this process. As a part of the PaRIS initiative [8], which recently field-tested the PaRIS-PQ in 18 countries [9], the NIPH followed the recommendations of the PaRIS Mental Health Group, which aims to harmonise PREMs and PROMs internationally [4, 10, 11]. Based on these guidelines, the five-item World Health Organization Well-Being Index (WHO-5) was selected for its strong validity, international relevance, and ease of integration into existing survey frameworks. It was used alongside two OECD-developed questions on overall life satisfaction and perceived meaning or purpose in life [10, 11].

The WHO-5 is one of the most widely used generic instruments for measuring subjective psychological well-being [12]. Originally developed for primary care, it has since been widely adopted in general population studies and across a range of clinical conditions, particularly for depression screening. The scale comprises five positively worded items rated on a Likert scale. It has been translated into over 30 languages and validated in many countries [13]. Its psychometric and clinical validity is well documented across age groups, diagnoses, and care settings, including mental health [12, 14–17]. Importantly, the WHO-5 is one of the few instruments that assesses well-being independently of physical or mental functioning [18].

Scores are obtained by summing the five items (range 0–25) and rescaling to 0–100, with higher values indicating better well-being. Widely used thresholds are ≤ 50, indicating reduced well-being warranting follow-up, and ≤ 28, suggestive of very low well-being [12]. These cut-offs are recommended for screening and monitoring purposes only, not for diagnostic classification [12]. Validation of the WHO-5 therefore concerns the interpretation of scores as indicators of subjective well-being and their intended use for monitoring change over time and comparing subgroups or services [9, 11, 13].

Most previous WHO-5 validation studies have been conducted in selected health care settings [12], with some also including general population samples [13, 19, 20]. The instrument has demonstrated good validity among psychiatric patients [21–23], and a study confirmed its robust psychometric properties in outpatient mental health care [24]. However, recent literature indicates that measurement properties of the WHO-5 are not entirely invariant across populations and settings [12, 13], and findings from psychiatric samples suggest that score distributions and cut-offs may vary [21, 23]. To date, little is known about its performance among patients recently discharged from specialised inpatient psychiatric care, a clinically vulnerable group with high symptom burden, comorbidity, and heterogeneous recovery trajectories. This may increase the risk of floor effects and reduced separation between mid-range categories. Moreover, few studies have validated the WHO-5 in nationwide samples. While nationwide validation studies are useful for examining subgroup differences in heterogeneous clinical populations, establishing population norms requires a representative population sample and is not attempted here. This study therefore aims to evaluate the reliability, construct validity, and measurement invariance of the WHO-5 in a sample of recently discharged adult psychiatric inpatients, using baseline data from a nationwide longitudinal survey.

## Methods

### Setting

In Norway, mental health services are organised to ensure continuity of care across specialised and community-based settings. Specialised services are primarily provided by public and private inpatient facilities under regional health authorities, which collaborate with municipalities to facilitate follow-up after discharge. Patients typically receive municipal care to support reintegration and long-term recovery after discharge. This model combines acute care with community-based support, in line with national guidelines that emphasise coordination to address patients’ varying needs and promote effective, continuous treatment [25–27].

### Data collection

The present study is part of the PRQMs-MAS programme (Research Council of Norway, project number 331891), which establishes a national longitudinal cohort of psychiatric inpatients followed up after discharge [5]. Baseline data were collected in January–February 2024. While the longitudinal design includes two follow-up assessments to measure PREMs and PROMs after discharge, only the baseline cross-sectional data are analysed in this study. The sample comprised all adult patients (≥ 18 years) discharged from specialised mental health facilities in Norway during a defined 3-month period (October–December 2023), covering both public and private institutions under contract with regional health authorities. In total, 8077 discharges were identified through the Norwegian Patient Registry (NPR), representing the full population of eligible discharges in the period. Of these, 85.4% (*n* = 6894) were registered users of the national digital portal Helsenorge and could therefore be invited to participate. The remaining 1183 (14.6%) were not contactable via this channel.

Invitations were sent electronically, with up to four SMS reminders to non-responders. Patients who completed the survey were eligible for the lottery draw of ten gift cards worth NOK 5000 each. Completed surveys were stored on the Services for Sensitive Data (TSD) platform at the University of Oslo and later transferred to NIPH’s secure environment.

### Questionnaire and outcome measures

The survey included 43 closed-ended and three open-ended questions, covering inpatient experiences, post-discharge services, current health and well-being, and background information. The questionnaire is available in Supplementary Material S1.

The section on health and well-being included the WHO-5, the OECD Assessment of Subjective Well-Being Core Items, and three self-rated health items. The WHO-5 comprises five positively worded items assessing well-being over the last two weeks: (1) “I have felt cheerful and in good spirits”; (2) “I have felt calm and relaxed”; (3) “I have felt active and vigorous”; (4) “I woke up feeling fresh and rested”; and (5) “My daily life has been filled with things that interest me”. Responses range from 0 (“At no time”) to 5 (“All the time”). Item scores (range 0–25) are summed and multiplied by four, yielding a total score from 0 to 100. The two OECD core items assessed subjective well-being [9]: (1) “Overall, how satisfied are you with life as a whole these days?”, and (2) “Overall, to what extent do you feel the things you do in your life are worthwhile?”, with responses on a scale from 0 (“Not at all”) to 10 (“Completely”). Self-rated mental health and general condition were assessed on a five-point scale from 1 (“Very poor”) to 5 (“Very good”), while self-reported physical health was rated on a five-point scale from 1 (“Excellent”) to 5 (“Poor”). These self-rated health items have previously been included in validated PREM instruments [6].

Three items from the Psychiatric Inpatient Patient Experience Questionnaire – Continuous Electronic Measurement (PIPEQ-CEM) outcome scale assessed whether the treatment had helped the patient to understand and cope with their mental health problems, and whether it had contributed to maintaining hope for a better life post-discharge [6]. Responses ranged from 1 (“not at all”) to 5 (“to a very large degree”). This enablement scale was used to test the convergent validity of the WHO-5.

Demographic variables included age, sex, education level, country of birth, previous psychiatric admissions, and employment status. Primary diagnoses and length of inpatient stays were obtained from the NPR, while education level and country of birth were obtained from Statistics Norway. Respondents were asked about participation in follow-up surveys, but only baseline data are used in this study.

### Statistical analysis

Descriptive statistics were used to examine item-level missing data, means, and floor effects for WHO-5 items. Floor effects, defined as the proportion of respondents selecting the lowest category (“At no time”), have typically been minimal in previous studies [12–14], and a proportion below 15% is generally acceptable [28]. However, higher levels may occur in clinical populations with severe psychological distress, potentially limiting sensitivity at low levels of well-being. In this study, floor effects were calculated per item and score distributions were inspected for clustering at the lower end, following established guidelines [12]. Given that the sample comprised recently discharged psychiatric inpatients, higher floor effects were expected. Descriptive statistics are presented for the WHO-5 total score, rescaled from 0 to 25 to a 0–100 scale for ease of interpretation and consistency with established cut-offs (≤ 50, ≤ 28), in line with WHO-5 practice. Each WHO-5 item was coded on its original 0–5 response scale, and all psychometric analyses (EFA, CFA, measurement invariance, and IRT) were conducted on these raw item scores. To assess potential selection bias, respondents and non-respondents were compared on available demographic variables (sex, age, and education) using chi-square (χ^2^) tests.

Construct validity and measurement invariance were assessed using exploratory factor analysis (EFA), confirmatory factor analysis (CFA), multi-group CFA analyses, hypothesis testing via correlations with theoretically related constructs, and item response theory (IRT). Cronbach’s alpha and McDonald’s ω were used to assess the internal consistency reliability of the WHO-5, with values ≥ 0.70 considered acceptable [28]. Item-deleted alpha values were examined to evaluate the contribution of each item.

EFA was estimated using minimum residual (minres) extraction based on a polychoric correlation matrix, treating the WHO-5 items as ordinal. Cases with missing values were handled by listwise deletion (< 1%), a commonly used approach when missingness is minimal. Factor retention was examined with parallel analysis and scree plot inspection [29]. In line with prior findings [19, 20, 30, 31], a one-factor solution was hypothesised. CFA was conducted to evaluate the one-factor model of the WHO-5, using weighted least squares with mean and variance adjustment (WLSMV) with ordered-categorical indicators and pairwise deletion (available-case) for missing data. Robust model fit was evaluated using the mean- and variance-adjusted chi-square (WLSMV χ^2^), the Comparative Fit Index (CFI), the Tucker–Lewis Index (TLI), the Standardised Root Mean Square Residual (SRMR), and the Root Mean Square Error of Approximation (RMSEA). Due to the sensitivity of χ^2^ to sample size, model evaluation relied primarily on CFI and TLI values ≥ 0.95, SRMR < 0.05, and RMSEA < 0.07, in line with established recommendations [32, 33]. RMSEA is reported with 90% confidence intervals, which is the default in lavaan and standard practice in CFA.

Measurement invariance was assessed through multi-group CFA across sex, age groups, and educational levels. These are socio-demographic variables commonly included in WHO-5 validation studies and emphasised in international PROM/PREM frameworks, standard sets, and implementations [12, 19, 20, 23, 34–36]. We used the same estimation specification as the single-group model: WLSMV; ordered-categorical indicators; pairwise deletion. Establishing invariance is critical to confirm that observed group differences reflect true variations in well-being rather than measurement artefacts. Invariance was tested at the configural, metric, and scalar levels, each involving increasingly strict constraints. Configural invariance assesses whether the same factor structure holds across groups; metric invariance tests equivalence of factor loadings; and scalar invariance examines equality of item thresholds. Model fit was assessed using CFI, RMSEA, and SRMR, while changes between models were evaluated relative to the less constrained model using recommended thresholds for decreases in CFI (ΔCFI ≤ 0.010) and increases in RMSEA (ΔRMSEA ≤ 0.015) [37–39]. DIFFTEST p-values from the WLSMV-adjusted χ^2^ difference test were computed for completeness but were not used as the primary decision criterion due to their sensitivity to sample size.

Convergent validity was evaluated by examining correlations between the WHO-5 and the OECD core items on subjective well-being. Based on previous research linking well-being to mental [19, 40–42] and physical health [43], we also included self-reported health measures. In addition, we assessed correlations with perceived treatment effects, understanding, coping, and hope, reflecting patient enablement [44]. We expected the WHO-5 to be associated with self-reported mental and physical health and treatment-related enablement, with the strongest correlation for mental health. As the physical health item was coded so that higher values indicate poorer health, we expected a negative correlation. Given non-normal data distribution, Spearman’s rank correlation was used.

IRT analyses were conducted to evaluate the psychometric properties of the WHO-5, assuming unidimensionality, that all items reflect a single underlying construct. Given the polytomous response format, we applied the graded response model (GRM) with marginal maximum likelihood (MML), which estimated item-specific discrimination (a) and threshold (b) parameters [45, 46]. Only respondents with all five WHO-5 items missing were excluded, while cases with partial missingness were retained, as the MML estimator incorporates available responses. Global model fit was evaluated using the M2 statistic with associated RMSEA and SRMSR (Standardised Root Mean Square of Residuals; the M2-based analogue of SRMR) [47]. Item performance was evaluated using the S–X^2^ item-fit statistic [48], supplemented with item-level RMSEA values, as well as discrimination and threshold parameters, which indicate how well items differentiate between levels of well-being and where response category boundaries are located along the latent trait continuum.

All analyses were conducted using IBM SPSS Statistics version 28 and R version 4.2.3 [49]. The psych package [50] was used for exploratory factor and reliability analyses, lavaan [51] and semTools [52] for confirmatory factor and measurement invariance analyses, and mirt [53] for item response theory modelling.

## Results

Respondent characteristics are shown in Table 1. A total of 2310 patients completed the survey (response rate: 33.5%). Most were women (64.0%), and 47.2% were aged 25–44 years. Regarding education, 31.9% had only primary education, and 33.3% held a university or college degree. The majority were born in Norway (84.9%), and 33.3% lived with a partner. Employment status varied: 23.6% were in paid work, while 53.6% were unable to work due to illness or poor health. Prior hospital experience also varied: 29.4% had never been admitted to psychiatric care, while 26.8% had been admitted five times or more. Length of stay ranged from 1 to 2 days (17.5%) to ≥ 1 month (16.8%). Before admission, 81.9% rated their mental health as “very poor” or “rather poor”, and 5.4% as “very good” or “rather good”. At survey time, 41.6% still rated their mental health as “very poor” or “rather poor”, while 18.3% reported it as “very good” or “rather good”. Current general condition was rated as “very poor” or “rather poor” by 36.6%, and “rather good” or “very good” by 25.9%. Physical health was rated “very good” or “excellent” by 13.5%, and “poor” or “rather good” by 57.0%.

**Table 1:** Respondent characteristics (*n* = 2310)

	*n*	%
Sex		
Female	1478	64.0
Male	832	36.0
Age, years		
18–24	304	13.2
25–44	1091	47.2
45–66	804	34.8
≥ 67	111	4.8
Education		
Primary school	725	31.9
Secondary school	791	34.8
University or college	757	33.3
Country/region of birth		
Norway	1957	84.9
Nordic countries (excl. Norway), EU/EFTA, UK, USA, Canada, Australia, New Zealand	164	7.1
Other countries (Europe outside EU/EFTA and UK, Africa, Asia, Americas excl. USA/Canada, Oceania excl. Australia/NZ, polar areas)	186	8.1
Married or living with a partner		
Yes	764	33.3
No	1528	66.7
Which of these terms best describes your current work situation?		
In paid employment (work for someone else)	546	23.6
Unable to work due to sickness or ill-health	1239	53.6
Not employed or looking for work	281	12.2
Self-employed (work for yourself)	61	2.6
Looking for work	145	6.3
Student or apprentice	186	8.1
Looking after the home	167	7.2
Retired	199	8.6
Other	258	11.2
Previous admissions		
0	676	29.4
1	389	16.9
2	274	11.9
3–5	343	14.9
> 5	617	26.8
Length of stay at this institution		
1 or 2 days	400	17.5
3–7 days	642	28.1
1–4 weeks	858	37.6
> 1 month	383	16.8
Self-perceived mental health prior to admission		
Very poor	1127	48.9
Rather poor	761	33.0
Neither good nor poor	291	12.6
Rather good	90	3.9
Very good	35	1.5
Self-perceived mental health		
Very poor	360	15.7
Rather poor	595	25.9
Neither good nor poor	919	40.1
Rather good	319	13.9
Very good	101	4.4
Current general condition		
Very poor	319	13.9
Rather poor	522	22.7
Neither good nor poor	862	37.5
Rather good	484	21.1
Very good	111	4.8
Self-perceived physical health		
Excellent	64	2.8
Very good	245	10.7
Good	680	29.6
Rather good	752	32.7
Poor	558	24.3

Respondents (*n* = 2310) differed from non-respondents (*n* = 4584) on sex, age, and education (Table S1). Women were somewhat more likely to respond than men, response rates were highest among those aged 25–66 and lowest among the youngest and oldest, and patients with higher education responded more often than those with primary education. These differences were statistically significant but moderate.

The distribution of responses and item-level mean scores are presented in Table 2. The proportion of missing responses on the WHO-5 items was low (0.6–0.8%). The mean total score on the WHO-5 was 33.4 (scale 0–100), indicating low well-being. A high proportion of lowest-category responses was observed for items 3 (“I have felt active and vigorous”) and 4 (“I woke up feeling fresh and rested”), with 28.2% and 35.0% of respondents selecting “at no time”, respectively. None of the items were excluded from further analysis. Item-level response distributions and mean scores are shown in Table 2 Subgroup differences by sex, age, and education are presented in Table S2, and score distributions are visualised in Fig. S1.

**Table 2:** Item-level responses and descriptive statistics for the WHO-5 (%)

	*N*	Missing	Mean*	At no time	Some of the time	Less than half of the time	More than half of the time	Most of the time	All the time
1. I have felt cheerful and in good spirits	2296	0.6	36.23	14.2	36.0	19.9	16.1	12.0	1.8
2. I have felt calm and relaxed	2292	0.8	36.11	17.7	33.0	19.0	15.0	12.0	3.3
3. I have felt active and vigorous	2296	0.6	30.86	28.2	28.6	18.2	13.3	9.4	2.4
4. I woke up feeling fresh and rested	2295	0.6	28.98	35.0	25.6	15.2	10.8	10.5	2.9
5. My daily life has been filled with things that interest me	2295	0.6	34.95	18.9	34.6	16.3	15.8	11.5	2.8
WHO-5 total score			33.44						

*Items were scored from 0 (“At no time”) to 5 (“All the time”) and transformed to a 0–100 scale for total and item-level mean scores. Higher scores indicate better well-being. “At no time” represents floor effect for items.

EFA using a polychoric correlation matrix and minimum residual extraction supported a unidimensional structure of the WHO-5. A single factor accounted for 71.7% of the total variance (eigenvalue = 3.59). Internal consistency was high (Cronbach’s α = 0.910, McDonald’s ω = 0.927), and item-deleted alpha values ranged from 0.881 to 0.899, indicating that all items contributed meaningfully to the scale’s reliability (Table 3).

**Table 3:** Factor loadings from exploratory factor analysis and reliability statistics

WHO-5 items	Factor loadings	Cronbach’s alpha/ McDonald’s ω	Cronbach’s alpha if item deleted
1. I have felt cheerful and in good spirits	0.897	0.910/0.927	0.881
2. I have felt calm and relaxed	0.828		0.893
3. I have felt active and vigorous	0.842		0.891
4. I woke up feeling fresh and rested	0.805		0.899
5. My daily life has been filled with things that interest me	0.859		0.887

**Fig. 1:**
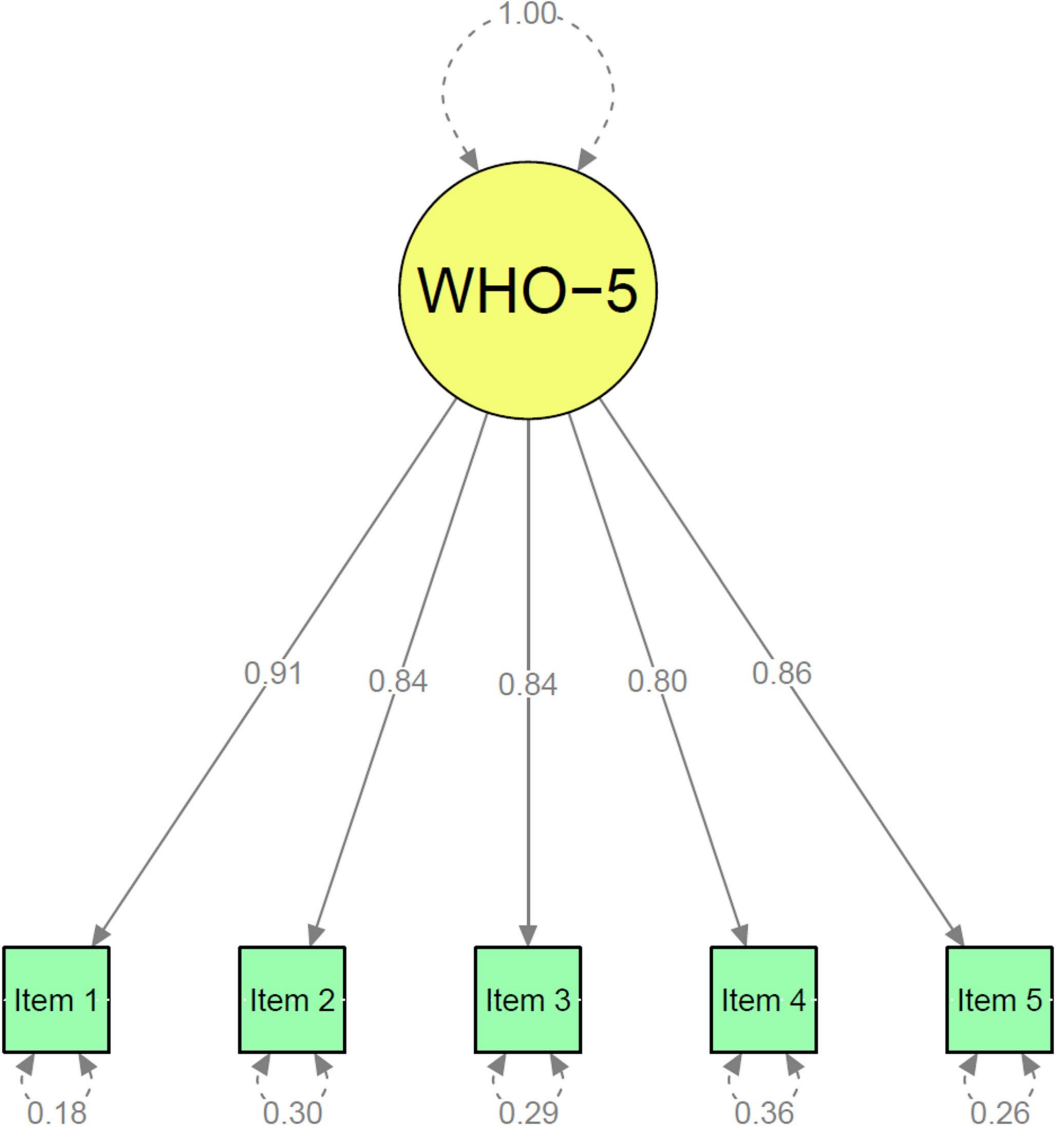
Confirmatory factor analysis for WHO-5. Numbers between WHO-5 and items show factor loadings. Numbers below items show residual variance (i.e., the proportion of item variance not explained by the latent factor)

The one-factor model was confirmed through CFA with the WHO-5 items specified as ordered categorical variables and estimated using WLSMV (Fig. 1). The χ^2^ test yielded χ^2^ (5) = 294.93, *p* < 0.001. Model fit indices were excellent for CFI (0.990), TLI (0.981), and SRMR (0.024), although the robust RMSEA was 0.149 (90% CI [0.136–0.163]), exceeding conventional thresholds. All item loadings were statistically significant (*p* < 0.001), with standardised values ranging from 0.802 to 0.905. R^2^ values ranged from 0.643 to 0.819, indicating substantial explained variance. Item 4 had the weakest factor loading, suggesting reduced association with the latent construct, but its removal did not improve model fit, including the RMSEA value.

Measurement invariance was supported across sex, age, and education (Table 4). Across models, CFI was high (0.990–0.994) and SRMR was low (≤ 0.033). RMSEA was high for the configural and metric models (0.129–0.163) but acceptable for the scalar models (0.041–0.052). Changes between nested models were small and within decision thresholds (ΔCFI = − 0.001–0.003; ΔRMSEA = − 0.090 to − 0.021). Although DIFFTEST *p*-values were often significant, decisions were based on change-in-fit criteria, supporting full measurement invariance and valid mean comparisons across subgroups.

**Table 4:** Fit indices for measurement invariance models of the WHO-5 across sex, age groups, and educational level

Model	Chi-square (χ^2^)	df	CFI	RMSEA	SRMR	ΔCFI	ΔRMSEA
Sex							
Configural	88.49	10	0.990	0.152	0.025	–	–
Metric	96.53	14	0.993	0.130	0.026	0.003	− 0.022
Scalar	136.96	33	0.993	0.052	0.025	0.000	− 0.077
Age groups							
Configural	106.24	20	0.992	0.163	0.027	–	–
Metric	168.26	32	0.994	0.142	0.033	0.002	− 0.021
Scalar	222.97	89	0.993	0.051	0.028	− 0.001	− 0.090
Educational level							
Configural	95.07	15	0.990	0.158	0.026	–	–
Metric	108.52	23	0.994	0.129	0.028	0.003	− 0.029
Scalar	139.43	61	0.994	0.041	0.026	0.001	− 0.088

ΔCFI and ΔRMSEA denote changes relative to the less constrained (previous) model (metric vs. configural; scalar vs. metric). Decision thresholds: ΔCFI ≤ 0.010; ΔRMSEA ≤ 0.015.

The WHO-5 correlated strongly with the OECD core items on subjective well-being, including overall life satisfaction (ρ = 0.764) and perceived meaning in life (ρ = 0.744) (Table 5). Similarly, strong correlations were found with self-perceived mental health (ρ = 0.727) and current general condition (ρ = 0.739). The correlation with self-rated physical health was moderate and negative (ρ = − 0.471), reflecting the item coding where higher scores indicate poorer health. Correlations with items on treatment-related understanding, coping, and hope were also significant, ranging from moderate to weak (ρ = 0.505–0.342).

**Table 5:** Correlations (Spearman’s rho) between WHO-5 and related constructs: subjective well-being (OECD core items), self-rated health, and perceived treatment outcomes

	ρ
Subjective well-being (OECD core items)	
Overall life satisfaction	0.764
Finding meaning or purpose in life	0.744
Self-rated health	
Self-perceived mental health	0.727
Current general condition	0.739
Self-rated physical health	− 0.471
Perceived treatment outcomes	0.471^1^
Treatment helped to understand mental health problems	0.342
Treatment helped to cope with mental health problems	0.463
Treatment helped to increase hope for a better life after discharge	0.505

^1^Composite score

IRT analyses provided further evidence of the scale’s performance. Discrimination parameters (a-values) ranged from 2.38 to 3.91, indicating variability in how well items differentiated between levels of well-being (Table 6). All estimated thresholds were ordered, indicating that the response categories functioned as intended. Some adjacent thresholds were located close to one another. Item 1 (“I have felt cheerful and in good spirits”) showed the highest discrimination (a = 3.91), reflecting high sensitivity to differences in the latent trait, while item 4 had the lowest (a = 2.38), indicating weaker differentiation. Threshold parameters (b-values) ranged from − 1.15 to 2.47, suggesting the items captured variation across low to moderate levels of well-being within this inpatient sample. Item 5 “My daily life has been filled with things that interest me” had widely spaced thresholds, whereas item 4 was more compressed, reflecting lower precision, particularly at the lower end of the well-being scale. Item fit was evaluated using the S–X^2^ statistic. Although all item tests were significant (*p* < 0.001), this is expected in large samples. Item-level RMSEA values ranged from 0.02 to 0.03, supporting acceptable item fit. Global model fit was acceptable: M2 (5) = 147.75, *p* < 0.001, RMSEA = 0.112 (90% CI [0.097–0.128]), SRMSR = 0.028.

**Table 6:** Parameter estimates (GRM) derived from IRT analysis of the WHO-5

	a	b1	b2	b3	b4	b5	S–X^2^	*p*
WHO-5								
1. I have felt cheerful and in good spirits	3.91	− 1.15	− 0.04	0.54	1.21	2.42	77.87	< 0.001
2. I have felt calm and relaxed	2.83	− 1.08	− 0.02	0.59	1.23	2.27	134.23	< 0.001
3. I have felt active and vigorous	2.86	− 0.70	0.15	0.76	1.42	2.44	131.96	< 0.001
4. I woke up feeling fresh and rested	2.38	− 0.50	0.29	0.84	1.39	2.47	98.54	< 0.001
5. My daily life has been filled with things that interest me	3.11	− 1.00	0.06	0.57	1.25	2.31	93.51	< 0.001
Range	2.38–3.91	− 1.15–− 0.50	− 0.04–0.29	0.54–0.84	1.21–1.42	2.27–2.47		

a = discrimination; b1–b5 = category thresholds; S–X^2^ = item-fit statistic; *p* < 0.05 indicates lack of fit

**Fig. 2:**
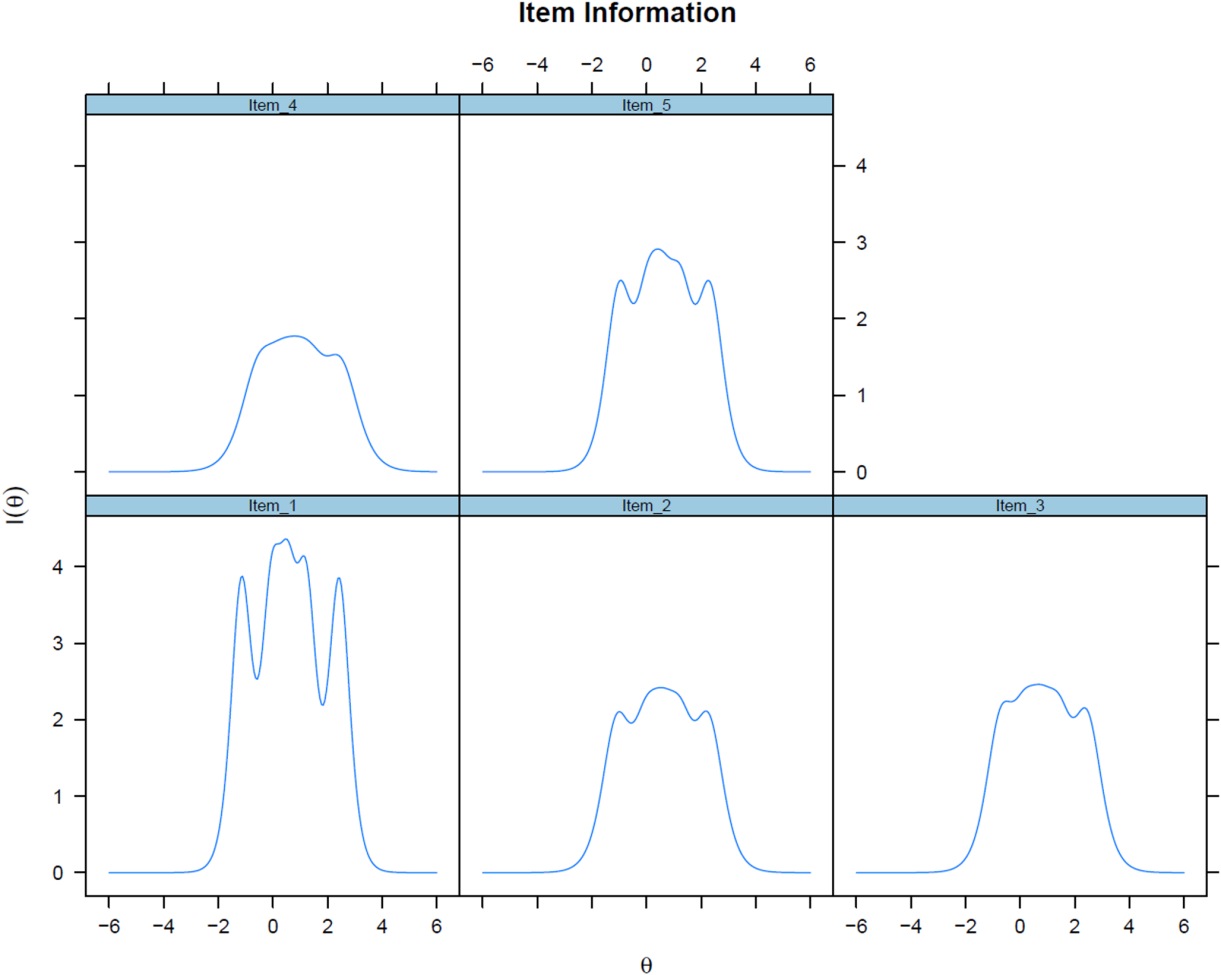
Item information functions for the WHO-5

Item information functions (Fig. 2) peaked around θ ≈ 0, reflecting the sample mean. Items 1 and 5 provided the most information overall due to higher discrimination (Table 6), while item 4 showed lower discrimination and more compressed thresholds. Consequently, item 4 contributed less total information, with modestly greater precision toward the lower end of the continuum. Thresholds were ordered for all items, but the middle thresholds (b2–b3, b3–b4) were closely spaced, leading to overlapping category curves across items.

**Fig. 3:**
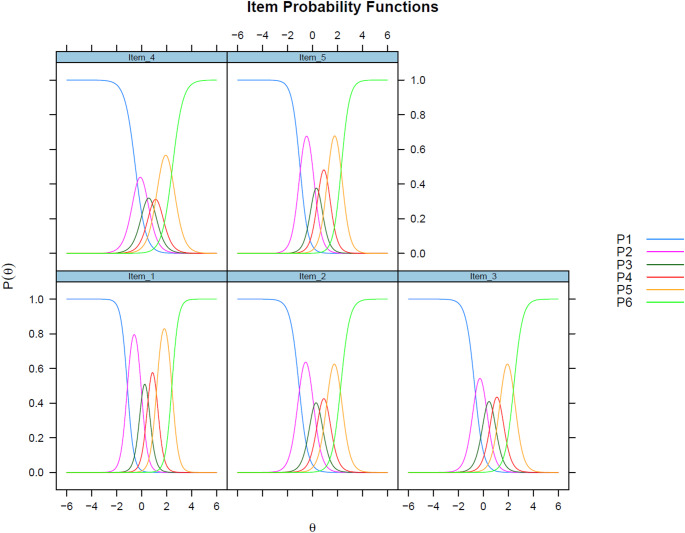
Categorical response curves for the WHO-5

Categorical response curves (Fig. 3) showed that response categories were generally ordered and captured a meaningful gradient of well-being. However, overlap was evident among the adjacent middle categories (2-4), particularly for item 4, where thresholds were closely spaced (b2 = 0.29, b3 = 0.84, b4 = 1.39). Although thresholds were ordered for all items (Table 6), the narrower spacing in the mid-range reduced resolution around moderate well-being.

## Discussion

This study assessed the psychometric properties of the WHO-5 in a large national sample of adults recently discharged from specialised inpatient mental health care. The results support the scale’s reliability and construct validity in this population, demonstrating high internal consistency, a clear unidimensional structure, and acceptable measurement invariance across sex, age, and educational level. Our findings align with prior validation studies [13, 21, 30, 31] and confirm the scale’s performance in a post-discharge psychiatric population, a context that has received limited attention. Moreover, the findings underscore the strengths of the WHO-5 as a brief, generic tool for assessing psychological well-being, well suited for clinical and research settings where time and cognitive demands on patients are key concerns.

The factor structure of the WHO-5 was supported by both EFA and CFA. The one-factor model explained 71.7% of the variance, with strong loadings and low residual variances for most items. Items 1 (“I have felt cheerful and in good spirits”) and 5 (“My daily life has been filled with things that interest me”) showed the strongest associations with the latent construct, while item 4 (“I woke up feeling fresh and rested”) contributed less and showed moderate to high floor effects. Although the RMSEA exceeded conventional thresholds, this may be due to the very low degrees of freedom of the five-item model rather than substantive misfit. RMSEA is known to overstate misfit in such cases [33], and more weight should therefore be placed on CFI, TLI, and SRMR, which indicated acceptable fit.

Measurement invariance findings suggest that the WHO-5 performs consistently across sex, age, and education groups, supporting valid mean comparisons. Configural, metric and scalar invariance were supported, indicating similar conceptualisation, stable item loadings, and comparable item thresholds across groups. These findings support the WHO-5 as a psychometrically sound and broadly applicable PROM in psychiatric care, in line with international standards for equitable cross-group measurement [4, 10, 11, 37].

Strong correlations between WHO-5 scores and measures of subjective quality of life and mental health support the scale’s concurrent validity. Significant but weaker associations with physical health and enablement further confirm its construct validity. These findings align with previous validation studies and reinforce the scale’s utility for monitoring recovery and well-being in psychiatric populations [13, 22, 23].

IRT analyses provided additional insight into item functioning. Discrimination values varied, with item 1 showing the strongest ability to distinguish between levels of well-being. In the GRM, θ is sample-centred by default, with zero representing the mean of the inpatient sample rather than a population-wide norm. This explains why the item information functions peaked at θ = 0 despite patients’ generally low absolute well-being compared with general populations. Within this sample, Items 1 and 5 were the most informative overall, while item 4, though lower in discrimination, provided slightly greater precision at lower levels. Across items, the close spacing of mid-range thresholds produced overlapping category curves and reduced resolution around moderate well-being, a limitation that is common in short PROMs. These findings mirror concerns raised in previous studies [12, 13] and may reflect challenges in capturing nuanced states of well-being after severe mental illness.

Moderate floor effects were observed for items 3 and 4, particularly item 4, where 35.0% of respondents selected the lowest response category. This aligns with previous findings in psychiatric populations, where well-being scores often skew toward the lower end. Previous findings on floor effects have been mixed, likely reflecting differences in study populations [21, 24]. These effects may reduce sensitivity to the level of deterioration at the very low end, since the lowest response category (“At no time”) already represents complete absence of well-being. This insensitivity is not unique to the WHO-5 but reflects a general limitation of brief, generic well-being instruments [12, 13]. In severely impaired populations, more condition-specific PROMs may be required to capture variation at the lowest levels of well-being. Although item 4 showed relatively low discrimination and compressed thresholds in IRT analyses, it still captured an aspect of well-being (feeling fresh and rested) that is not fully represented by the other items. This conceptual contribution supports its retention despite weaker statistical performance. Thresholds spanned a reasonable range on the latent continuum, supporting measurement across low to moderate levels of well-being in this clinical sample. As θ in IRT is sample-centred, item information functions naturally peak around the sample mean rather than a population norm. The observed spectrum thus reflects the clinical characteristics of recently discharged psychiatric inpatients. This implies that sensitivity is reduced at the higher end, and limited precision in the mid-range of the well-being continuum.

The current response categories of WHO-5 are somewhat ‘unbalanced’, i.e., the theoretical distance between the two poorest (“At no time”, “Some of the time”) and the two best categories (“All the time”, “Most of the time”) are different. For all WHO-5 items, 50–60% of patients in our study clustered in the two poorest response categories. Given the clinical severity of mental health inpatients and the unbalanced response categories, there might be room for further differentiating low levels of well-being by introducing an additional category between the two poorest categories (“Rarely/seldom”). Thus, we propose further experimental research in this setting by randomizing patients to either receive the original 6-category format or an alternative response format more adjusted to the patient group (e.g., a 7-category format with “Rarely/seldom), as refinements of response options have been shown to improve measurement precision [54]. A revised format could be relevant and useful for applications involving patient populations with poor mental health, facilitating a more fine-grained measurement of the lowest levels of well-being and the ability to measure differences between individuals/groups and over time.

Including PROMs like the WHO-5 alongside established PREMs supports a more holistic understanding of care quality. A recently published scoping review highlighted the paucity of studies that concurrently assess PREMs and PROMs in psychiatric and substance use disorder services [55]. This dual approach, endorsed by the OECD’s PaRIS initiative [4, 8–11], reflects international priorities and enables health systems to align patient perspectives on both care experiences and treatment outcomes. The use of standardised instruments like the WHO-5 facilitates international benchmarking, while also offering locally relevant data to support service improvement [4, 8–11, 34, 35].

The WHO-5 can be applied at both the individual and system levels. At the individual level, it supports screening and monitoring of well-being at discharge, with thresholds of ≤ 50 and ≤ 28 indicating reduced and very low well-being, respectively [12]. Given the 0–100 scaling, differences occur in steps of four points. A change of around 10 points (typically 8–12 points) is commonly regarded as clinically meaningful [12]. At the service and system level, aggregated WHO-5 data can be reported as mean scores with confidence intervals, distributions, and proportions below the 50-point threshold, ideally with case-mix adjustment. This approach is consistent with evidence from large-scale validations [13] and aligns with international initiatives such as the OECD PaRIS programme, which promotes the integration of PROMs and PREMs in quality monitoring [4, 8–11]. While the WHO-5 is not diagnostic and may show floor or ceiling effects, it provides a valid and efficient indicator of psychological well-being that can complement PREMs, other PROMs, and clinical outcomes [12, 24, 36, 55].

### Strengths and limitations

This study is among the first to psychometrically validate the WHO-5 in a large, post-discharge psychiatric population. The nationwide sampling frame enhances breadth and heterogeneity for subgroup analyses; furthermore, the estimates could be regarded as preliminary WHO-5 reference values for this patient group, but with uncertain generalizability because of the low response rate. The WHO-5 demonstrated high internal consistency, strong construct validity, and acceptable levels of measurement invariance across key demographic groups. Its brevity, clarity, and positive phrasing make it especially suitable for routine use in mental health services, including digital platforms and routine follow-up systems.

Integrating PROMs like the WHO-5 into existing PREMs frameworks enables a more comprehensive understanding of care quality, aligning patient experiences with outcomes that matter to service users. This approach reflects international policy priorities and facilitates both local service development and cross-national comparisons through standardised measurement [34, 35].

Despite its strengths, several limitations should be noted. Of the 8077 eligible patients discharged during the study period, 6894 (85.4%) were invited via the national digital platform Helsenorge, while 1183 (14.6%) were not contactable via this channel. Among those invited, 2310 completed the WHO-5, corresponding to a 33.5% response rate and 28.6% of the total eligible population. This two-step reduction, first due to lack of contactability and then non-response, reduced the final analytic sample. Comparison of respondents and non-respondents showed systematic but moderate demographic differences: women, middle-aged, and higher educated patients were somewhat more likely to respond, whereas response rates were lower among the youngest and oldest groups. These differences suggest some potential for selection bias, particularly regarding well-being estimates, as mean scores and proportions may not fully generalise to the target population. However, they are unlikely to have substantially biased the psychometric evaluation of the WHO-5, which primarily concerns internal structure and measurement properties. This is supported by prior validation studies showing robust factor structure and invariance across demographic subgroups despite selective response patterns (e.g., 12, 13, 23, 56).

Findings should also be interpreted in light of the Norwegian context, where the use of Helsenorge as the sole recruitment channel may have excluded certain groups (e.g., older adults, those with lower education, limited digital literacy, or immigrant backgrounds). While this limits generalisability to other countries and health systems, the study nonetheless draws on a large, nationwide sample of recently discharged inpatients across diverse institutions and diagnostic categories. The response rate is comparable to other large-scale mental health surveys and observed respondent–non-respondent differences were moderate (see Table S1), supporting the robustness of the psychometric findings despite some limitations in representativeness. Furthermore, increasing focus on PROMs (including well-being, WHO-5) in international mental health system quality measurement and evaluation, e.g., through OECD’s PaRIS initiative [4, 10, 11], means that our validation study has relevance across a range of countries.

While prior studies report limited non-response bias in mental health surveys [56, 57], these typically focus on care experiences, not outcomes. Additionally, the large sample size increased sensitivity to minor model misfit in CFA and IRT analyses. These factors should be considered in interpreting the results.

Floor effects and overlapping response categories may reduce the scale’s sensitivity at the very low end of well-being, particularly among patients with severe psychological distress.

Finally, important contextual factors such as diagnostic diversity, treatment-related experiences, and patients’ perspectives on admission were not analysed here, as the present paper focused specifically on the psychometric evaluation of the WHO-5. These aspects will be examined in future research based on the broader cohort datasets.

## Conclusions

The WHO-5 demonstrated strong reliability and construct validity as a measure of psychological well-being among patients discharged from specialised mental health care. Despite some limitations, including moderate floor effects and overlapping mid-range response categories, the WHO-5 remains a robust and accessible instrument. To enhance precision, future revisions might explore refining the response format or supplementing the WHO-5 with condition-specific PROMs to better capture low levels of well-being.

Integrating PROMs like the WHO-5 alongside established PREMs can support a more holistic assessment of mental health care quality, aligning patients’ experiences with outcomes that matter to them. While this dual approach is a key international priority [4, 8–11, 35, 36], evidence in psychiatric populations remains limited. Our findings demonstrate the relevance of using the WHO-5 alongside PREMs in this context, thereby adding to the emerging evidence base. Future work should aim to refine PROMs such as the WHO-5 to ensure greater sensitivity to diverse patient needs while also supporting broader system evaluations and international benchmarking. Such integration can strengthen mental health services’ ability to deliver patient-centred care that captures both experiences and outcomes.

## Supplementary Information

Below is the link to the electronic supplementary material.

Supplementary Material 1: English version of the baseline questionnaire.

Supplementary Material 2

Supplementary Material 3

Supplementary Material 4

## Data Availability

The data sets generated and/or analysed during the current study are not publicly available due to the need to protect personal data, but anonymous data are available from the corresponding author on reasonable request.
