# Supplementary material for: Psychometric performance of the WHO-5 well-being index in a nationwide sample of inpatients discharged from specialised mental health care
Source: Qual Life Res. 2025 Dec 29;35(1):16. doi: 10.1007/s11136-025-04104-9 (PMC12748132; doi:10.1007/s11136-025-04104-9)
Supplement: Supplementary file 1 — Supplementary Material 1: English version of the baseline questionnaire. [file 11136_2025_4104_MOESM1_ESM.pdf]

Norwegian Institute of Public Health

What are your experiences with your inpatient stay in mental health care and the time after discharge?

We would like to hear about your experiences from the institution where you were recently admitted, your experiences with various services after discharge, and how you are currently experiencing your health. The purpose of the survey is to improve services for patients in mental health care.

Waiting time and admission

1. Was your admission to this institution planned in advance or an emergency admission?

- ☐ Planned
- ☐ Emergency

2. Did you have to wait for admission?

- ☐ No
- ☐ Yes, but not long
- ☐ Yes, quite long
- ☐ Yes, far too long
- ☐ *Not applicable*

3. Were you admitted against your will?

- ☐ Yes
- ☐ No

4. Do you find that the admission was necessary or unnecessary?

- ☐ Very unnecessary
- ☐ Somewhat unnecessary
- ☐ Neither/nor
- ☐ Somewhat necessary
- ☐ Very necessary

5. How would you describe your mental health prior to this admission?

- ☐ Very poor
- ☐ Quite poor
- ☐ Neither poor nor good
- ☐ Quite good
- ☐ Very good

Therapists and staff

Keep the therapists and staff at the institution in mind when you answer the following questions.

6. Did you have enough time for discussions and contact with the therapists/staff?

- ☐ Not at all
- ☐ To a small extent
- ☐ To some extent
- ☐ To a large extent
- ☐ To a very large extent
- ☐ *Not applicable*

7. Did you find that the therapists/staff understood your situation?

- ☐ Not at all
- ☐ To a small extent
- ☐ To some extent
- ☐ To a large extent
- ☐ To a very large extent
- ☐ *Not applicable*

Involvement at the institution

8. Did you find that the treatment was adapted to your situation?

- ☐ Not at all
- ☐ To a small extent
- ☐ To some extent
- ☐ To a large extent
- ☐ To a very large extent
- ☐ *Not applicable*

Involvement at the institution

9. Was the treatment during this stay voluntary or did you feel forced to receive it?

- ☐ Completely voluntary
- ☐ Somewhat voluntary
- ☐ Neither voluntary nor involuntary
- ☐ Somewhat involuntary
- ☐ Completely involuntary
- ☐ *Not applicable*

Environment and activities

10. Did you feel safe at the institution?

- ☐ Not at all
- ☐ To a small extent
- ☐ To some extent
- ☐ To a large extent
- ☐ To a very large extent
- ☐ *Not applicable*

11. Was the range of activities available at the institution satisfactory?

- ☐ Not at all
- ☐ To a small extent
- ☐ To some extent
- ☐ To a large extent
- ☐ To a very large extent
- ☐ *Not applicable*

Other assessments

12. Did the help and treatment you received at the institution help you better *understand* your mental health issues?

- ☐ Not at all
- ☐ To a small extent
- ☐ To some extent
- ☐ To a large extent
- ☐ To a very large extent
- ☐ *Not applicable/don't know*

Other assessments

13. Did the help and treatment you received at the institution help you better *cope* with your mental health issues?

- ☐ Not at all
- ☐ To a small extent
- ☐ To some extent
- ☐ To a large extent
- ☐ To a very large extent
- ☐ *Not applicable/don't know*

14. Did the help and treatment you received at the institution give you confidence that life would be better after discharge?

- ☐ Not at all
- ☐ To a small extent
- ☐ To some extent
- ☐ To a large extent
- ☐ To a very large extent
- ☐ *Not applicable/don't know*

---

15. Overall, was the help and the treatment you received at the institution satisfactory?

- ☐ Not at all
- ☐ To a small extent
- ☐ To some extent
- ☐ To a large extent
- ☐ To a very large extent

16. Overall, to what extent did you benefit from the treatment at the institution?

- ☐ No benefit
- ☐ Small benefit
- ☐ Some benefit
- ☐ Large benefit
- ☐ Very large benefit

Other assessments

17. Do you think the length of this stay was appropriate?

- ☐ Yes
- ☐ No, too short
- ☐ No, too long

Feel free to write more about your experiences from your inpatient stay here:

____

____

Experiences with health services after discharge

The following questions concern your experiences with health services you have received for your mental health issues and overall health after being discharged from inpatient mental health care.

Please provide an overall assessment of these services.

18. Do you discuss with health professionals involved in your care what is most important for you in managing your mental health issues and wellbeing?

- ☐ Yes, definitely
- ☐ Yes, to some extent
- ☐ No, not really
- ☐ No, definitely not
- ☐ *Don't know*
- ☐ *Not applicable*

19. Are you involved as much as you want to be in decisions about your wellbeing and the services you receive?

- ☐ Yes, definitely
- ☐ Yes, to some extent
- ☐ No, not really
- ☐ No, definitely not
- ☐ *Don't know*
- ☐ *Not applicable*

20. Are you considered as a "whole person" rather than just a disease/condition in relation to your wellbeing and the services you receive?

- ☐ Yes, definitely
- ☐ Yes, to some extent
- ☐ No, not really
- ☐ No, definitely not
- ☐ *Don't know*
- ☐ *Not applicable*

21. Were there times when you had to repeat information that should have been in your care records?

- ☐ Yes, definitely
- ☐ Yes, to some extent
- ☐ No, not really
- ☐ No, definitely not
- ☐ *Don't know*
- ☐ *Not applicable*

22. Is your health care organized in a way that works for you?

- ☐ Yes, definitely
- ☐ Yes, to some extent
- ☐ No, not really
- ☐ No, definitely not
- ☐ *Don't know*
- ☐ *Not applicable*

23. Do you have a single professional who takes responsibility for coordinating your care across the services that you use?

- ☐ Yes
- ☐ No
- ☐ I do not receive services from more than one provider
- ☐ *Don't know*

24. Do you have a care plan that takes into account all your needs in relation to your mental health issues and overall health?

- ☐ Yes
- ☐ No
- ☐ *Don't know*

25. Do you have enough support from your healthcare professionals to help you manage your mental health issues and overall health?

- ☐ I do not need support
- ☐ I have had no support
- ☐ I sometimes have enough support
- ☐ I often have enough support
- ☐ I always have enough support
- ☐ *Don't know*
- ☐ *Not applicable*

26. To what extent do you receive useful information when you need it to help you manage your mental health issues and overall health?

- ☐ I do not receive any information
- ☐ I sometimes receive enough information
- ☐ I often receive enough information
- ☐ I always receive enough information
- ☐ I receive too much information
- ☐ *Don't know*
- ☐ *Not applicable*

27. How confident are you that you can manage your mental health issues and overall health?

- ☐ Very confident
- ☐ Confident
- ☐ Somewhat confident
- ☐ Not confident at all
- ☐ *Not applicable*

Feel free to write more about your experiences with health services after being discharged here:

Municipal services and NAV

28. Have you received help from the municipality or NAV (Norwegian Labour and Welfare Administration) after being discharged from the institution?

- ☐ Yes (go to question 29)
- ☐ No (go to question 30)

29. From which services have you received help?

30. Overall, to what extent do you feel that you have received the help you need from the municipality or NAV?

- ☐ Not at all
- ☐ To a small extent
- ☐ To some extent
- ☐ To a large extent
- ☐ To a very large extent
- ☐ *Not applicable*

Feel free to write more about your experiences with help from the municipality or NAV here:

Your health and quality of life

31. Overall, how satisfied are you with life as a whole these days?

The following question asks how satisfied you feel, on a scale from 0 to 10. Zero means you feel "not at all satisfied" and 10 means you feel "completely satisfied".

- ☐ 0 Not at all satisfied
- ☐ 1
- ☐ 2
- ☐ 3
- ☐ 4
- ☐ 5
- ☐ 6
- ☐ 7
- ☐ 8
- ☐ 9
- ☐ 10 Completely satisfied

Your health and quality of life

32. Overall, to what extent do you feel the things you do in your life are worthwhile?

The following question asks how worthwhile you feel the things you do in your life are, on a scale from 0 to 10. Zero means you feel the things you do in your life are "not at all worthwhile", and 10 means "completely worthwhile".

- ☐ 0 Not at all worthwhile
- ☐ 1
- ☐ 2
- ☐ 3
- ☐ 4
- ☐ 5
- ☐ 6
- ☐ 7
- ☐ 8
- ☐ 9
- ☐ 10 Completely worthwhile

33. How would you describe your *mental* health?

- ☐ Very poor
- ☐ Rather poor
- ☐ Neither good nor poor
- ☐ Rather good
- ☐ Very good

34. Overall, how are you feeling today?

- ☐ Very poor
- ☐ Rather poor
- ☐ Neither good nor poor
- ☐ Rather good
- ☐ Very good

35. How would you describe your *physical* health?

- ☐ Excellent
- ☐ Very good
- ☐ Good
- ☐ Rather good
- ☐ Poor

By answering the questions below, you can give us an overall picture of how well or poorly you have been feeling lately.

Please choose the response that best describes how you have felt for most of the past two weeks.

36. I have felt cheerful and in good spirits

- ☐ All of the time
- ☐ Most of the time
- ☐ More than half of the time
- ☐ Less than half of the time
- ☐ Some of the time
- ☐ At no time

37. I have felt calm and relaxed

- ☐ All of the time
- ☐ Most of the time
- ☐ More than half of the time
- ☐ Less than half of the time
- ☐ Some of the time
- ☐ At no time

38. I have felt active and vigorous

- ☐ All of the time
- ☐ Most of the time
- ☐ More than half of the time
- ☐ Less than half of the time
- ☐ Some of the time
- ☐ At no time

39. I woke up feeling fresh and rested

- ☐ All of the time
- ☐ Most of the time
- ☐ More than half of the time
- ☐ Less than half of the time
- ☐ Some of the time
- ☐ At no time

Your health and quality of life

40. My daily life has been filled with things that interest me

- ☐ All of the time
- ☐ Most of the time
- ☐ More than half of the time
- ☐ Less than half of the time
- ☐ Some of the time
- ☐ At no time

A little about you

41. Have you been admitted to a psychiatric institution before this admission?

- ☐ No
- ☐ Yes, once
- ☐ Yes, twice
- ☐ Yes, 3–5 times
- ☐ Yes, more than 5 times

42. Are you married or living with a partner?

- ☐ Yes
- ☐ No

43. Which of the following best describes your current situation?

You can select more than one option.

- ☐ In paid work (employed by someone)
- ☐ Unable to work due to illness or poor health
- ☐ Not working and not seeking employment
- ☐ Self-employed
- ☐ Job seeker
- ☐ Student or apprentice
- ☐ Homemaker
- ☐ Pensioner
- ☐ Other
- ☐ Don't know

Would you like to contribute to more knowledge about how patients are doing over time?

We would like to learn more about how patients are doing after being discharged from inpatient mental health care. We therefore invite you to complete up to two additional questionnaires about your experiences with different services and your health. You will receive the questionnaires 2–8 months after completing this one.

Would you be willing to answer up to two additional questionnaires about your experiences with different services and your health?

- ☐ Yes, I would like to participate
- ☐ No, I do not wish to participate

If you would like to learn more about the project, you can read more here: (https://www.fhi.no/en/cristin-projects/ongoing/a-new-generation-of-patient-reported-quality-measurements-in-mental-health-/). You may withdraw your consent at any time, see the contact information provided in the information letter.

Thank you for taking the time to complete the survey!

NIPH
Norwegian Institute of Public Health
