# Supplementary material for: Psychometric performance of the WHO-5 well-being index in a nationwide sample of inpatients discharged from specialised mental health care
Source: Qual Life Res. 2025 Dec 29;35(1):16. doi: 10.1007/s11136-025-04104-9 (PMC12748132; doi:10.1007/s11136-025-04104-9)
Supplement: Supplementary file 2 — Supplementary Material 2 [file 11136_2025_4104_MOESM2_ESM.docx]

**Supplementary file S2**

**Table S1:** Comparison of respondents (n = 2,310) and non-respondents (n = 4,584) by sex, age, and education.

| **Subgroup** | **Respondents %** | **Non-respondents %** | **χ² (df)** | **p-value** |
| --- | --- | --- | --- | --- |
| **Sex** |  |  |  |  |
| Women | 39.2 | 60.8 | 122.3 (1) | <0.001 |
| Men | 26.6 | 73.4 |  |  |
| **Age** |  |  | 55.0 (3) | <0.001 |
| 18–24 | 28.7 | 71.3 |  |  |
| 25–44 | 32.6 | 67.4 |  |  |
| 45–66 | 39.2 | 60.8 |  |  |
| ≥67 | 25.3 | 74.7 |  |  |
| **Education** |  |  | 157.7 (2) | <0.001 |
| Primary school | 26.1 | 73.9 |  |  |
| Secondary school | 34.5 | 65.5 |  |  |
| University or college | 44.3 | 55.7 |  |  |

Percentages are row percentages within each demographic category.
