# Supplementary material for: Psychometric performance of the WHO-5 well-being index in a nationwide sample of inpatients discharged from specialised mental health care
Source: Qual Life Res. 2025 Dec 29;35(1):16. doi: 10.1007/s11136-025-04104-9 (PMC12748132; doi:10.1007/s11136-025-04104-9)
Supplement: Supplementary file 3 — Supplementary Material 3 [file 11136_2025_4104_MOESM3_ESM.docx]

**Supplementary file S3**

**Table S2:** Descriptive statistics for WHO-5 scores across demographic subgroups.

| **Subgroup** |  | **Mean WHO-5 score** | **Standard deviation (SD)** |  | **Skewness** | **Kurtosis** |
| --- | --- | --- | --- | --- | --- | --- |
| **Gender** |  |  |  |  |  |  |
| Female |  | 31.78 | 22.99 |  | 0.74 | -0.18 |
| Male |  | 36.39 | 24.96 |  | 0.52 | -0.65 |
| **Age** |  |  |  |  |  |  |
| 18–24 |  | 27.72 | 21.68 |  | 1.02 | 0.65 |
| 25–44 |  | 31.79 | 22.28 |  | 0.70 | -0.12 |
| 45–66 |  | 35.84 | 24.78 |  | 0.54 | -0.66 |
| ≥67 |  | 48.31 | 28.97 |  | -0.10 | -1.34 |
| **Education** |  |  |  |  |  |  |
| Primary school |  | 29.21 | 23.03 |  | 0.91 | 0.26 |
| Secondary school |  | 34.16 | 24.50 |  | 0.60 | -0.59 |
| University or college |  | 36.59 | 23.03 |  | 0.56 | -0.50 |
