# Supplementary figures and images for: Psychometric performance of the WHO-5 well-being index in a nationwide sample of inpatients discharged from specialised mental health care
Source: Qual Life Res. 2025 Dec 29;35(1):16. doi: 10.1007/s11136-025-04104-9 (PMC12748132; doi:10.1007/s11136-025-04104-9)

**Supplementary file S4**

**Figure S1:** Item-level histograms for WHO-5.

**
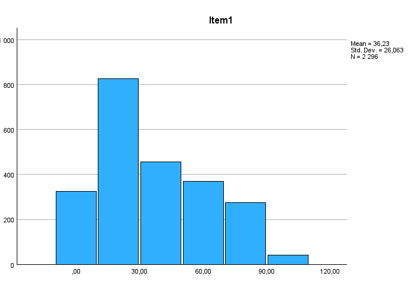
**

**
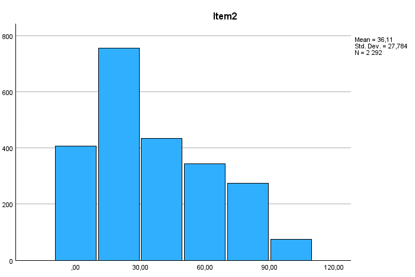
**

**
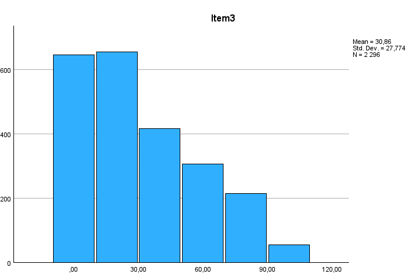
**

**
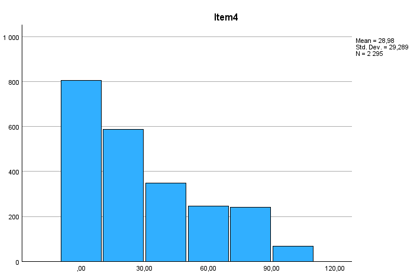
**

**
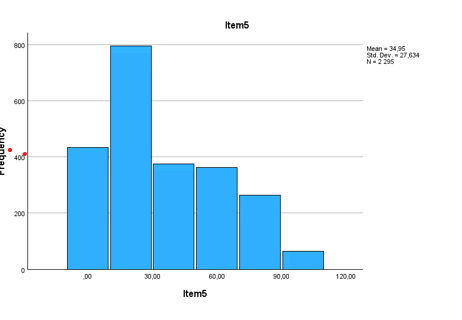
**

Supplement: Supplementary file 4 — Supplementary Material 4 [file 11136_2025_4104_MOESM4_ESM.docx]
